# Effect of caffeine on muscle oxygen saturation during short-term all-out exercise: a double-blind randomized crossover study

**DOI:** 10.1007/s00394-022-02875-2

**Published:** 2022-04-02

**Authors:** Carlos Ruiz-Moreno, Jorge Gutiérrez-Hellín, Beatriz Lara, Juan Del Coso

**Affiliations:** 1 Exercise Physiology Laboratory, Camilo José Cela University, Madrid, Spain; 2 Faculty of Health Sciences, Universidad Francisco de Vitoria, Madrid, Spain; 3 Centre for Sport Studies, Rey Juan Carlos University, Fuenlabrada, Spain

**Keywords:** Anaerobic test, Exercise performance, Ergogenic aid, Dietary supplement, Adenosine

## Abstract

**Purpose:**

The ergogenic effect of oral caffeine administration on short-term all-out exercise performance is well established. However, the potential mechanisms associated with caffeine’s ergogenicity in this type of exercise are poorly understood. The aim of this study was to investigate whether caffeine intake modifies muscle oxygen saturation during the 15-s Wingate Anaerobic Test.

**Methods:**

Fifteen moderately trained individuals (body mass = 67.4 ± 12.3 kg; height 171.3 ± 6.9 cm; age 31 ± 6 years) took part in two identical experimental trials after the ingestion of (a) 3 mg/kg of caffeine or (b) 3 mg/kg of cellulose (placebo). After 60 min for substances absorption, participants performed a 15-s Wingate test on a cycle ergometer against a load representing 7.5% of participant’s body mass. Muscle oxygen saturation was continuously measured during exercise with near-infrared spectroscopy and blood lactate concentration was measured 1 min after exercise.

**Results:**

In comparison to the placebo, the oral administration of caffeine increased peak power by 2.9 ± 4.5% (from 9.65 ± 1.38 to. 9.92 ± 1.40 W/kg, *P* = 0.038; effect size (ES), 95% confidence intervals = 0.28, 0.05–0.51), mean power by 3.5 ± 6.2% (from 8.30 ± 1.08 to 8.57 ± 1.12 W/kg, *P* = 0.044; ES = 0.36, 0.01–0.71) and blood lactate concentration by 20.9 ± 24.7% (from 12.4 ± 2.6 to 14.8 ± 4.0 mmol/L, *P* = 0.005; ES = 0.59, 0.16–1.02). However, caffeine did not modify the curve of muscle oxygen desaturation during exercise (lowest value was 23.1 ± 14.1 and 23.4 ± 14.1%, *P* = 0.940).

**Conclusion:**

Caffeine’s ergogenic effect during short-term all-out exercise seems to be associated with an increased glycolytic metabolism with no influence of enhanced muscle oxygen saturation.

## Introduction

Caffeine (1,3,7-trimethylxanthine) is a potent stimulant of the central nervous system used in nearly all sports disciplines to enhance physical performance [1]. Recent meta-analyses reveal that oral caffeine intake, in a dose of 3-to-9 mg per kg of body mass, increases performance in endurance [2], short-term high-intensity exercise [3], as well as in other forms of exercise that combine repeated efforts and pauses [4, 5]. The ample effect of caffeine on exercise performance seems to be associated with the nature of the compound and its several mechanisms of action, as explained in detail elsewhere [6]. Briefly, caffeine is rapidly absorbed after oral intake in the stomach and intestine [7] and the lipophilic nature of this substance allows its transition through the blood–brain barrier [8]. In the brain, caffeine possesses the ability to inhibit the fatiguing effects of adenosine by blocking adenosine-specific receptors. In turn, the blockade of adenosine-specific receptors by caffeine causes the inability of adenosine to stimulate fatigue while increasing the release of norepinephrine, dopamine, acetylcholine, serotonin, among other neurotransmitters [9].

Although the blockage of adenosine receptors is the main mechanism associated with caffeine’s ergogenic activity during exercise [10], other physiological actions of caffeine may contribute to enhanced physical performance. Caffeine is sufficiently hydrophobic to pass through all biological membranes and is readily distributed throughout all tissues of the body [7]. Increased muscle oxygen saturation [11], improved function of the Na + /K + pump [12], enhanced calcium ion release from the sarcoplasmic reticulum [13] and increased fatty acid mobilization and fat oxidation [14] have been proposed as contributors for increased performance after oral caffeine intake. Interestingly, these mechanisms are mainly effective during endurance-based activities as caffeine has an amplified ergogenic effect on more oxidative fibre types [15]. However, the mechanisms associated with the ergogenic effect of caffeine on short-term all-out exercise are poorly understood.

Enhanced anaerobic metabolism with caffeine intake has been proposed as a plausible explanation of caffeine’s effects on anaerobic exercise. However, peak lactate concentration after supramaximal efforts is present in some [16–18] but not all experiments [19, 20]. To the authors’ knowledge, no previous study has investigated if enhanced muscle oxygen saturation may contribute to the ergogenic benefit of caffeine during short-term all-out exercise, a mechanism already found in aerobic exercise [11]. The caffeine-induced increase of muscle oxygen saturation may be a mechanism to explain caffeine’s ergogenicity as the contribution of aerobic metabolism is relevant for efforts longer than 10 s [21]. Hence, this study aimed to investigate whether caffeine intake modifies muscle oxygen saturation during the 15-s Wingate Anaerobic Test, to ultimately unveil the potential contribution of muscle oxygen saturation to the ergogenic effect of caffeine during short-term all-out exercise. We hypothesized that oral administration of caffeine would increase muscle oxygen saturation during the 15-s Wingate test, indicating enhanced oxygen availability in the muscle contributes to caffeine’s ergogenicity during short-term all-out exercise.

## Methods

### Participants

Fifteen healthy and moderately trained (> 4 days of training/week; > 45 min/day) participants took part in this research voluntarily. All participants were enrolled in high-intensity intermittent exercise training (*e.g.,* team sports, cycling and triathlon), including at least one exercise session with short-duration near-to-maximal-intensity bouts per week. They had a mean ± standard deviation (SD) body mass of 67.4 ± 12.3 kg; height of 171.3 ± 6.9 cm; age of 31 ± 6 years. There were eight men and seven women in the study sample. Women were always tested in the luteal phase to standardize data collection although recent data indicate that women obtain similar ergogenic benefits from oral caffeine administration across the menstrual cycle [22]. An a priori sample size calculation indicated that at least ten participants were required to obtain statistically significant differences between caffeine and placebo on Wingate peak cycling power. The required sample size was calculated to obtain a caffeine-induced increase of 0.35 W/kg based on a previous study that reported such benefit in men and women with 3 mg/kg of caffeine [23]. The sample size was calculated using the formula proposed by Vincent and Weir [24] with a statistical power of 0.80 and a two-tailed α level of 0.05. As criteria for inclusion, participants had to be non-smokers with an age between 18 and 40 years, report no previous cardiopulmonary disease in their clinical history, have had no musculoskeletal injuries in the 3 months before the onset of the investigation, low caffeine consumption (< 1 mg/kg/day) as per the categorization proposed by Filip et al. [25]. The exclusion criteria included the use of medications in the previous month and allergy to caffeine. In women, the use of oral contraceptives was an exclusion criterion. One week before the onset of the experiments, participants were fully informed of the experimental process, and they were asked to sign a written informed consent to participate. The study was approved by the ethics committee of the Camilo José Cela University and complied with the latest version of the Declaration of Helsinki in 2013.

### Experimental design

A randomized, double-blind, placebo-controlled and crossover experimental design was used in this investigation. All the participants performed two identical experimental trials separated by at least 76 h to allow substance elimination of the substance and full recovery. Participants ingested either (a) 3 mg of caffeine per kg of body mass (100% purity, Bulk Powders, United Kingdom) or 3 mg of cellulose per kg of body mass (Guinama, Spain). The substances were administered in identical opaque capsules with 150 mL of tap water and 60 min before the onset of trials. Neither the participants nor the researchers had any information about the assignment of the substances to each trial. To produce a double-blind experiment, a researcher who was not involved in the data collection of the experiment prepared the placebo and caffeine capsules and assigned the order of the trials in a random manner using a research randomizer software (www.randomizer.org). This same researcher provided the capsule assigned to each trial to the participants and certified the ingestion of the capsule. All tests were conducted in a laboratory setting to ensure constant ambient conditions (21.5 ± 0.3 ºC and 45 ± 2% relative humidity).

### Familiarization and standardizations

After enrollment, participants were encouraged to avoid nutritional supplements and sympathetic stimulants in their diets and to maintain their training routines during the whole duration of the experiment. One week before the first experimental trial, participants attended twice to familiarize themselves with the 15-s Wingate anaerobic test and the remaining study procedures. On those days, their body mass (± 50 g, Radwag, Poland) was measured to calculate substances dosage in an individualized manner and skinfold thickness was measured on the *vastus lateralis* of both legs (left limb = 5.6 ± 1.8 mm; right limb 5.6 ± 2.3 mm) to assure that muscle oxygen saturation could be accurately measured [26]. The day before the first experimental trial, participants were advised to abstain from all caffeine-containing foods, not to drink alcohol, perform a light exercise session and adopt a standardized diet and fluid intake. Fluid and diet guidelines [27, 28] were given to assure carbohydrate bioavailability and euhydration in all experimental trials. Specifically, the diet was customized to the preferences and allergies of each participant and provided at least 7 g/kg of carbohydrate in the day before the trial, and at least 2 g/kg of carbohydrate in the meal the morning of the experiment. Participants were also encouraged to ingest 500 mL of water two hours before arriving at the laboratory. The food and drinks ingested in the 24 h before the first trial were recorded in a diary and they were replicated before the second trial.

### Experimental trials

All trials were performed in the morning to avoid the effect of circadian variations in the results of the study. Participants arrived at the laboratory at 9.00 am, 3 h after their last meal. Upon arrival, participants received the assigned capsule in an unidentifiable plastic bag and swallowed the capsule with water. Researchers verified the ingestion of the capsule. Then, a near-infrared spectroscope (NIRS; Moxy, Fortiori Design LLC, Minnesota, USA) was positioned longitudinally in the *vastus lateralis* of each leg, three cm above the patella and midway between the trochanters and the lateral epicondyle of the femur. The NIRS was used to assess muscle oxygen saturation with a frequency of 0.5 Hz. The *vastus lateralis* was chosen because of its key contribution to power production during pedaling [29]. This device has good reliability for the assessment of local oxygen saturation during exercise [29]. The position of the NIRS devices was carefully replicated between tests. Each NIRS device was firmly attached to the skin with a tubular net to ensure that the device would not swing on the leg during exercise. Then, participants rested on a stretcher in a supine position until 60 min had passed from the oral ingestion of the substances to reach near-to-maximal plasma caffeine concentration [7].

After the resting period, participants performed a 10-min warm-up at 50–100 W followed by several short sprints with a light load. Then, participants performed an adapted version of the Wingate test on a cycle ergometer (SNT Medical, Cardgirus, Spain) with a duration of 15 s and against a load that represented 7.5% of the participant's body mass. We selected the 15-s version of the Wingate test, instead of the more traditional 30-s version, because our objective was to investigate the effect of caffeine on exercise performance with peak anaerobic contribution. In this case, peak ATP-PC and glycolytic contributions during the Wingate test are obtained during the first 15 s of the test [21].

All participants were informed about the need of fulfilling a series of compulsory premises to accept the test as valid: (a) start in a stationary position (b) with the dominant leg ready to pedal (c) and maintaining contact with the saddle during the whole test. Participants were told that they had to pedal as fast as they could for the 15 s and the command “stop” was given to participants 1 s after the end of the test to assure that participants pedaled at maximal intensity during the whole test. During the test, standardized encouragement was given to the participants by the same researcher who was blinded to the treatments. This researcher also verified that participants remained seated during the whole test. During the Wingate test, cycling power output was measured with a frequency of 1 Hz. The highest value of cycling power was considered as peak cycling power and the average of cycling power for the duration of the trial was considered as mean cycling power.

After the end of the test, participants continued pedaling at 50 W for 5 min and a blood sample was obtained from a fingertip just one minute after the end of the Wingate test to analyze blood lactate concentration (Lactate pro-2, Arkay, Japan). Five minutes after the end of the test, participants were asked about their self-perceived exertion using the traditional Borg scale (from 6 to 20 arbitrary units) and about their feelings of muscle power during exercise (from 1–10 arbitrary units).

### Statistical analysis

The results of each trial were blindly introduced into the statistical package SPSS v 27.0 for analysis. Normality was tested and confirmed for each variable with the Shapiro–Wilk test (all variables *P* > 0.05). Muscle oxygen saturation of the dominant and non-dominant limbs were averaged as the between-limb difference was < 1.7 ± 3.0% at all time-points during exercise. For the variables measured once during each trial (*e.g.,* peak and mean cycling power, blood lactate concentration, self-perceived exertion and muscle power), differences between caffeine and placebo trials were identified with paired t-tests. For the variables measured several times in each trial (*i.e.,* muscle oxygen saturation), differences between caffeine and -placebo trials were identified with a two-way analysis of variance (ANOVA). For each variable, the main effects of substance and time, in addition to the substance × time interaction, were calculated with Bonferroni corrections. The effect size (ES) ± 95% confidence intervals (CI) was also calculated in all caffeine-placebo comparisons by using Cohen’s *d* units [30]. The magnitude of the effect size was interpreted as follow: less than 0.2 = trivial; between 0.2 and 0.6 = small; between 0.6 and 1.2 = moderate; between 1.2 and 2.0 = large; between 2.0 and 4.0 = very large; higher than 4.0 = extremely large [31]. The significance level was set at *P* < 0.050 and all data are presented as means ± SD for the whole group of participants.

## Results

**Fig. 1:**
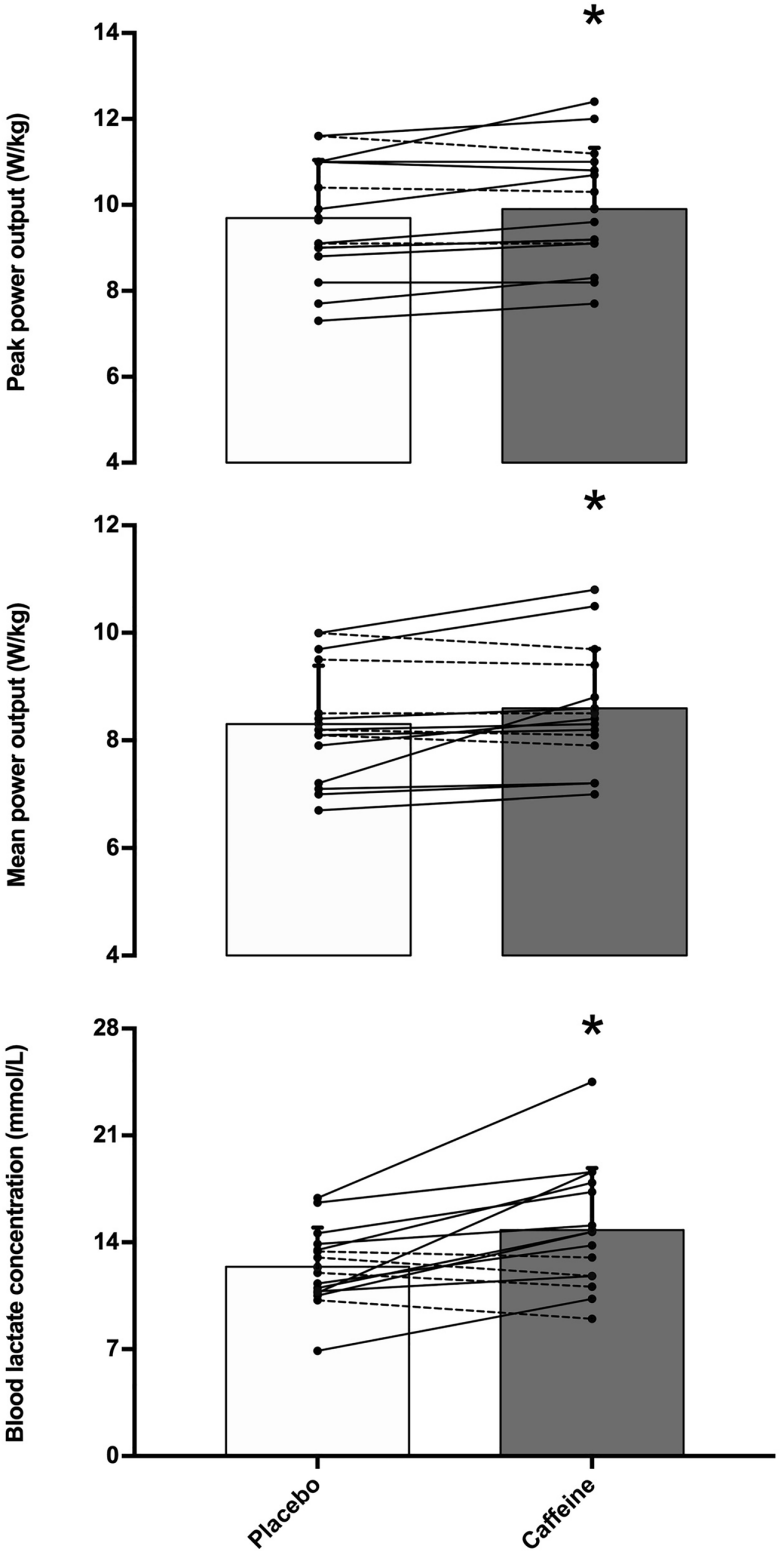
Peak cycling power output, mean cycling power output and post-exercise blood lactate concentration during a 15-s Wingate test with the oral administration of 3 mg/kg of caffeine or a placebo. (*) Caffeine different from placebo at *P* < 0.050. The bars represent mean values for 15 moderately trained participants. Each line represents values with placebo vs caffeine for each participant; continuous lines depict participants with values with caffeine with respect to placebo and the dashed lines depict individuals with values with caffeine with respect to placebo

There were main effects of substance (*F* = 4.76, *P* = 0.047) and time (*F* = 15.37, *P* < 0.001) on cycling power during the 15-s Wingate test, with no interaction between these effects (*F* = 0.73, *P* = 0.655). In comparison to the placebo, the oral administration of caffeine increased peak cycling power by 2.9 ± 4.5% (from 9.65 ± 1.38 to. 9.92 ± 1.41 W/kg, *P* = 0.038; ES, 95% CI = 0.28, 0.05–0.51) and mean cycling power by 3.5 ± 6.2% (from 8.30 ± 1.08 to 8.57 ± 1.12 W/kg, *P* = 0.044; ES = 0.36, 0.01–0.71, Fig. 1). There was a main effect of time on muscle oxygen saturation (*F* = 25.24, *P* < 0.001) but the main effect of substance did not reach statistical significance (*F* = 1.98, *P* = 0.183) with no interaction between these factors (*F* = 0.38, *P* = 0.888). The ingestion of caffeine did not affect the lowest value of muscle oxygen saturation measured during exercise (23.1 ± 14.1 and 23.4 ± 14.1%, for placebo and caffeine, respectively; *P* = 0.940). Post-exercise blood lactate concentration increased by 20.9 ± 24.7% with the oral administration of caffeine (from 12.4 ± 2.6 to 14.8 ± 4.0 mmol/L, *P* = 0.005; ES = 0.59, 0.16–1.02, Fig. 1). Self-reported values of exertion in the Borg scale were unaffected by the administration of caffeine (19.3 ± 1.3 and 19.3 ± 0.8 arbitrary units for placebo and caffeine, respectively; *P* = 0.80). Their perceived force–velocity was also unaffected by caffeine (8.4 ± 0.8 vs. 8.7 ± 0.9 arbitrary units, *P* = 0.43).

## Discussion

The aim of this study was to investigate whether caffeine intake modifies muscle oxygen saturation during the 15-s Wingate Anaerobic Test. The ultimate goal of this investigation was to assess the role of muscle oxygen saturation as a potential mechanism for caffeine’s ergogenicity during short-term all-out exercise. The main findings of this investigation indicate that oral administration of 3 mg/kg of caffeine was effective to increase peak and mean cycling power during a 15-s Wingate test. Caffeine also increased post-exercise blood lactate concentration, but muscle oxygen saturation remained unaltered with the intake of this stimulant. Collectively, this information suggests that caffeine’s ergogenic effect during short-term all-out exercise seems to be associated with an enhanced glycolytic metabolism with no influence on muscle oxygen saturation.

The conflicting results of the first experiments investigating the effect of oral administration of caffeine on the Wingate test [18, 32] are still present in more recent investigations [33, 34]. The lack of an ergogenic effect of caffeine on the Wingate test in some investigations is unclear as in these investigations the participants’ fitness level and the dose of caffeine administered were not different from those investigations with confirmed caffeine’s ergogenicity in the Wingate test [18, 32–35] It is possible that habituation to caffeine -due to chronic intake of this substance [36]-, and the identification of caffeine intake -to obtain psychological advantages produced by this stimulant [37], -, may explain some of the differences between investigations. Still, the cause of lack of caffeine’s ergogenicity in some investigations remains uncertain. Recent evidence suggests that the ergogenic effect of caffeine on Wingate performance may be of similar magnitude in men and women [23], although it may be progressively reduced with chronic ingestion of the substance [38]. The current investigation suggests an ergogenic effect of caffeine for the 15-s Wingate test with an improvement of 2.9 and 3.5% on peak and mean cycling power, respectively (Fig. 1). The ergogenic effect of caffeine was present in both men and women (change in mean power: 3.8% in men and 3.1% in women; change in peak power: 2.3% in men and 3.6% in women) and it was likely aided by the low habitual caffeine intake of the participants of this investigation. However, in the current investigation, there were some individuals who did not present increased cycling performance with caffeine intake. Specifically, 3 out of 15 individuals did not increase peak power with caffeine and 5 out of 15 individuals did not increase mean cycling power. The lack of ergogenic benefit of caffeine in some individuals during the Wingate test may be associated with within-individual variability and the fact that there was only one caffeine–placebo comparison performed in the current experiment. When the effect of caffeine on the Wingate test is tested several times in moderately trained individuals, all individuals respond to some extent to caffeine [39], challenging the hypothesis of the existence of non-responders to caffeine. Hence, it is still possible that those individuals who did not increase cycling performance with 3 m/kg of caffeine in the current experiment could have presented an ergogenic response to this substance with higher doses of caffeine or if they had performed the Wingate test over multiple treatment days.

Another possible source of between-individual variability on the response to caffeine intake during the Wingate test is associated genetics, particularly in candidate genes associated with caffeine metabolism. Investigations on the two main polymorphisms linked to the interindividual differences in response to caffeine intake (*i.e.,* − 163C > A in the *CYP1A2* gene [40] and 1976C > T in the *ADORA2A* gene [41]) revealed that individuals with different genotypes in these polymorphisms obtained similar benefits of caffeine on the Wingate test. Additionally, a recent investigation tested the effect of 14 genetic polymorphisms on the response to caffeine intake during the Wingate test and the authors concluded that variations in these candidate genes did not modify the effect of caffeine on the Wingate test [33]. However, these results of this latter investigation may have been influenced by the lack of ergogenic effect of caffeine on Wingate performance [33]. To date, most of the current evidence indicates that genetic variability in genes associated to caffeine metabolism does not modify caffeine's benefits on short-duration maximal-intensity exercise [33, 40]. Still, some studies indicate the contrary for endurance exercise [42]. Collectively, all this information suggests that acute caffeine intake is, overall, an effective supplementation protocol to enhance cycling power during the Wingate test. The magnitude of the ergogenic effect of caffeine may present some interindividual differences and some individuals do not obtain exercise performance benefits from caffeine intake. The interindividual differences in response to caffeine may be more associated with the within-individual variability during testing rather than an effect of caffeine dose, training level or genetics, as the ergogenic effect of caffeine has been found in a wide range of caffeine doses, participants’ fitness levels and with different genetic profiles.

**Fig. 2:**
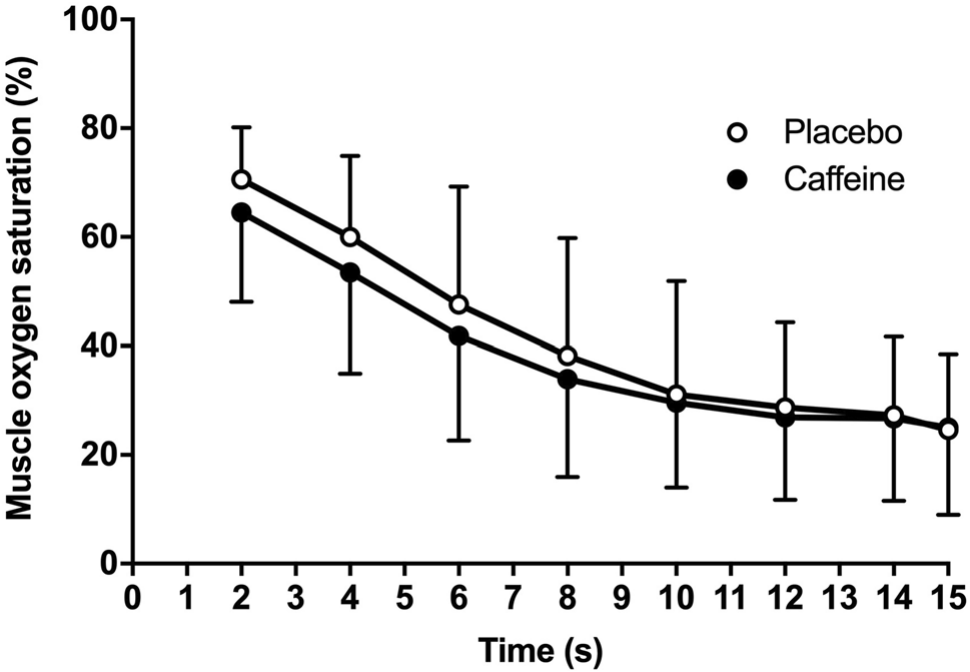
Muscle oxygen saturation during a 15-s Wingate test with the oral administration of 3 mg/kg of caffeine or a placebo. Data are mean ± standard deviation for 15 moderately trained participants

Despite the evident ergogenic benefit obtained with caffeine intake, muscle oxygen saturation in the *vastus lateralis* remained unaltered through the testing in the caffeine trial. In the placebo trials, muscle oxygen saturation changed from baseline values of ~ 75% to ~ 23% by the end of the 15 s of all-out exercise, while this pattern was almost identical with caffeine. These data are in line with other forms of lower limb exercise until fatigue [43, 44] suggesting that volitional interruption of high-intensity exercise succeeds even when muscle oxygen saturation is well above 0%. More importantly, the reduction of muscle oxygen saturation reveals the contribution of aerobic metabolism to energy production during the 15-s Wingate test. Although ATP-PC (for the first 5 s) and glycolysis (from 5 s for up to 15 s) are the main metabolic pathways to produce power during the Wingate test [21], aerobic metabolism provides ~ 20% of total power production and oxygen uptake (VO_2_) is 70% of the individual’s peak oxygen uptake (VO_2peak_) at the 15 s time point of the Wingate test [45]. The contribution of aerobic metabolism can reach 35% of the total power production, and VO_2_ can be 90% of VO_2peak_ in the last stages of the 30-s Wingate test [21]. Despite the evident aerobic contribution to power production in the different versions of the so-called Wingate Anaerobic Test and the evidence that acute caffeine intake increases muscle oxygen saturation during exercise [11], the current investigation reveals that enhanced muscle oxygen saturation was not a mechanism for the ergogenic benefit of caffeine during the 15-s Wingate test. This is because the reduction of muscle oxygen saturation followed a similar pattern in the caffeine and placebo trials (Fig. 2). This means that other mechanisms are behind caffeine’s benefits for short-term all-out exercise. However, it is still needed to investigate if enhanced muscle oxygen saturation is a mechanism associated with caffeine’s ergogenicity in longer high-intensity exercise protocols, as the 30-s Wingate test.

Interestingly, post-exercise blood lactate concentration was higher after the caffeine intake than with the placebo, suggesting a higher role of glycolytic metabolism to power production during the Wingate test. Higher blood lactate concentration after the Wingate test is a recurrent finding [18, 22, 38], although there are some other investigations in which post-exercise blood lactate concentration remained similar to the placebo [34, 46]. During the Wingate test, energy from the glycolysis explains 83% and 81% of the variance of peak and mean cycling power, while additions of energy from ATP-PC and aerobic metabolism do not improve the explanation of variance of peak and mean cycling power [18]. This means that glycolysis is the key metabolic element for the cycling power values obtained during the Wingate test. Additionally, the concomitant increase in power output and post-exercise blood lactate concentration with oral caffeine administration suggests that enhanced glycolysis is associated with caffeine’s ergogenicity in the Wingate test. Of note, the increase of glycolytic activity during exercise after caffeine ingestion may likely be related to stimulation of the central nervous system through blockage of adenosine receptors [47] rather than a local effect of caffeine within the active muscle. This would explain why participants obtained higher cycling performance and higher post-exercise blood lactate concentration levels with caffeine despite similar values of perceived exertion, in comparison to the placebo trial.

This research presents several limitations that should be discussed. First, the NIRS devices were located on the *vastus lateralis* of both legs. Although this muscle is a key contributor to power production during cycling [29] it is still possible that the effect of caffeine on muscle oxygen saturation would be different in other leg muscles. Second, we selected the dose of 3 m/kg of caffeine to test the effect of this stimulant on muscle oxygen saturation because this same dose has been effective to enhance muscle oxygen saturation during a ramp exercise test [11]. Although 3 mg/kg did not modify muscle oxygen saturation during the 15-s Wingate tests, higher doses of caffeine may induce different responses on local oxygen saturation. Third, we used the 15-s modified version of the Wingate Anaerobic test due to its high-reliability levels and because it has been found that 3 mg/kg of caffeine can increase cycling performance in this test [22, 23, 38]. However, the effect of caffeine on muscle oxygen saturation may be different in other forms of anaerobic exercise such as the 30-s Wingate Anaerobic test. Last, we included women and men in the sample of participants because we have recently found that the magnitude of the ergogenic effect of caffeine on the 15-s Wingate test is of similar magnitude in both sexes [23]. However, further investigation should test whether caffeine similarly affects muscle oxygen saturation during exercise in men and women, particularly as there may be some between-sex differences in caffeine metabolism [48].

In conclusion, the oral administration of 3 mg/kg of caffeine increased peak and mean cycling power during the 15-s Wingate test. The magnitude of the ergogenic effect of caffeine (2.9–3.5%) suggests that this substance confers important benefits for short-term all-out exercise. The caffeine-induced increased cycling performance was accompanied by higher post-exercise blood lactate concentration while muscle oxygen saturation remained unaltered through the test. These outcomes suggest that caffeine’s ergogenic effect on the 15-s Wingate test is primarily associated with enhanced glycolysis with no influence of muscle oxygen saturation.

For those willing to use caffeine as an ergogenic aid for short-term all-out performance, caffeine, in the form of pills, should be ingested at least 45 min prior to exercise to allow peak serum caffeine concentration during exercise [7] (although some sources of caffeine such caffeinated chewing gums may allow faster caffeine absorption [49]). The ergogenic effect of caffeine is obtained in doses ranging between 3 and 9 mg/kg. However, the authors of this study recommend the use of 3 mg/kg of caffeine to obtain performance benefits as the frequency and magnitude of caffeine adverse effects are higher with 9 mg/kg of caffeine [50]. The use of an accurate dose of caffeine in mg per kg of participant’s body mass is probably more important than the source of caffeine. Still, the use of supplements with pure caffeine instead of multi-ingredient supplements/foods (*e.g.,* pre-workouts, energy drinks, coffee, etc.) may avoid possible interactions of caffeine with other substances. Avoiding habituation to caffeine may be a wise option to maximize the magnitude of the potential benefit of acute caffeine intake on exercise performance [38]. Therefore, caffeine should be used occasionally during the training week, selecting the use of this substance before high-intensity training days or competitions. Finally, the risk/benefit of caffeine supplementation should be assessed individually, and special care should be taken when evaluating the use of caffeine in young athletes and individuals with low fitness levels.
